# Construction of exosome-related genes risk model in kidney cell carcinoma predicts prognosis and immune therapy response

**DOI:** 10.18632/aging.205767

**Published:** 2024-05-07

**Authors:** Chao Gao, Wei Huang, Qiang Su, Jingxian Li, Wei Wang, Yuanjiong Qi, E Du, Zhihong Zhang

**Affiliations:** 1 Tianjin Institute of Urology, The Second Hospital of Tianjin Medical University, Tianjin, China

**Keywords:** RCC, exosome, prognosis, machine learning, clinical model

## Abstract

Renal cell carcinoma (RCC) is one of the most prevalent types of urological cancer. Exosomes are vesicles derived from cells and have been found to promote the development of RCC, but the potential biomarker and molecular mechanism of exosomes on RCC remain ambiguous. Here, we first screened differentially expressed exosome-related genes (ERGs) by analyzing The Cancer Genome Atlas (TCGA) database and exoRBase 2.0 database. We then determined prognosis-related ERGs (PRERGs) by univariate Cox regression analysis. Gene Dependency Score (gDS), target development level, and pathway correlation analysis were utilized to examine the importance of PRERGs. Machine learning and lasso-cox regression were utilized to screen and construct a 5-gene risk model. The risk model showed high predictive accuracy for the prognosis of patients and proved to be an independent prognostic factor in three RCC datasets, including TCGA-KIRC, E-MTAB-1980, and TCGA-KIRP datasets. Patients with high-risk scores showed worse outcomes in different clinical subgroups, revealing that the risk score is robust. In addition, we found that immune-related pathways are highly enriched in the high-risk group. Activities of immune cells were distinct in high-/low-risk groups. In independent immune therapeutic cohorts, high-risk patients show worse immune therapy responses. In summary, we identified several exosome-derived genes that might play essential roles in RCC and constructed a 5-gene risk signature to predict the prognosis of RCC and immune therapy response.

## INTRODUCTION

Renal cell carcinoma (RCC) originates from the epithelial cells of the renal tubules and accounts for approximately 90% of renal cancers [1]. It has a mortality rate of nearly 30-40% and brings about a large number of deaths per year. The aetiology of RCC is complex, including gender, obesity, hypertension, smoking, chronic kidney disease, and so on [2]. Recent research has reported that the incidence of RCC is increasing and causes large-scale public health problems [3]. Renal clear cell carcinoma (KIRC), the most common type of renal cell carcinoma, accounts for about 75% of all renal cell carcinomas [4]. Although clear cell carcinoma detected early can be successfully cured surgically, metastasis still occurs in up to 30% of patients, and the prognosis of this metastatic cancer is extremely poor; it seems to be a common problem in urological malignancy [5–7]. For these metastatic clear cell carcinomas, systemic therapy based on immunosuppressive agents or targeted drug therapy is an option [8], but their therapeutic efficacy remains limited. Therefore, exploring new potential markers for prognostic prediction and individualized therapy is of great clinical importance.

Exosomes are extracellular vesicles with a diameter of about 30–150 nm [9]. Almost all cells in the body can secrete exosomes, including mesenchymal stem cells, tumour cells and immune cells [10]. Depending on the origin of the cell, exosomes contain a variety of substances, including nucleic acids, lipids, as well as metabolic and cell surface proteins [11]. Exosomes have a variety of activities, such as remodelling the extracellular matrix and transmitting signals and molecules to other cells [12]. Previous studies have shown that tumour-derived exosomes play a role in regulating tumour immunity and are essential for the formation and metastasis of the pre-metastatic niche (PMN) [13]. For instance, exosomes derived from breast and prostate cancer cells have been found to induce tumours by transferring their miRNAs [14]. Exosome miR-200 from metastatic breast cancer cells could enhance epithelial-mesenchymal transformation (EMT) and metastasis [15]. In addition, exosome substances stem from different cancer cells, such as nucleic acids, signalling proteins, and metabolites, and could irritate the tumour-promoting effects of stromal cells [16]. Fibroblast-derived exosomes could promote breast cell metastasis by inducing Wnt-PCP autocrine signalling [17]. In renal clear cell carcinoma, research has reported exosomes could promote metastasis and other tumour progression events [18, 19]. For example, RCC-derived exosomes facilitate tumour development by inducing macrophage polarization via transferring lncARSR; cancer stem cells (CSC) - derived exosomes transported miR-19b-3p into CCRCC cells and initiated EMT-promoting metastasis. Therefore, exploring the relationship between exosomes and renal clear cell carcinoma is necessary for further developing potential therapeutic targets based on exosomes. So far, research has begun to explore the exosome-related potential biomarkers in kidney cancer. Yoshino et al. found that exosomal MYO15A could be a diagnostic and therapeutic target in RCC [20]; He et al. established and validated novel exosomal mRNA-based signatures for the early detection of ccRCC and differential diagnosis of uncertain renal masses [21]. However, few studies exploit the potential exosome-related biomarkers based on large-scale RNA-sequence data and construct exosome-related clinical models for predicting RCC’s diagnosis and therapeutic effects.

In our study, we tried to explore potential exosome-related genes by utilizing large-scale RNA-sequence data from TCGA and exoRbase databases. Based on Cox regression analysis and multiple machine learning methods, we identified a 5-gene risk signature called prognosis-related exosome-related genes (PRERGs) signature to predict the prognosis of KIRC. External independent datasets (E-MTAB-1980 and TCGA-KIRP) proved that the risk signature is practical. Further, tumour immune infiltration analysis and external immunotherapeutic datasets revealed that the risk signature could predict the effects of patient-received immune therapy. We hope that these organized data can provide a theoretical basis for further experimental research based on exosomes in KIRC.

## MATERIALS AND METHODS

### Data acquisition

Exosome-related data containing 15 KIRC and 118 healthy samples were downloaded from exoRBase database (http://www.exorbase.org/). The mRNA expression profile and clinical data of the TCGA-KIRC dataset, including 535 tumour tissues and 72 normal tissues, and TCGA-KIRP, including 285 tumour tissues, were downloaded from the public website (https://xena.ucsc.edu/public). The TCGA-KIRC and KIRP datasets’ mRNA expression values were transformed into log2(TPM+1) units. E-MTAB-1980 dataset containing 101 RCC samples was downloaded from the Array Express database (https://www.ebi.ac.uk/arrayexpress). E-MTAB-1980 is an external RCC data that contains clinical data and normalized mRNA expression data. Four immune therapy-related datasets, GSE135222 (*n* = 27), GSE78820 (*n* = 28), GSE79671 (*n* = 36), and GSE42664 (*n* = 45), were downloaded from the Gene Expression Omnibus (GEO) database, respectively (https://www.ncbi.nlm.nih.gov/geo/).

### Differential expression analysis

Differential expression analysis was performed using the limma R package to screen for differentially expressed genes (DEGs). |log2(Fold change)| >1 and *P*-value < 0.05 were used as filtering thresholds for DEGs.

### Survival analyses, gDS, and target development levels (TDLs)

Based on the survival R package, the mRNA expression data matched with clinical survival data was used to conduct uni-variable Cox regression analysis. The risk regression model assessed the predictive effect to identify the prognosis-related genes. The ideal cut-off value of gene expression was calculated by the surv_cutpoint R function from the survminer R package. Then, the data was divided into the high- and low-expression groups. Kaplan-Meier survival curves were depicted to show the prognostic difference between the high- and low-expression groups. gDS score of each gene in kidney cancer cells was obtained from the CCLE database (https://sites.broadinstitute.org/ccle/) and was used to demonstrate the importance of each gene for cells. Each gene’s PubTator score and target development levels were obtained from a public website (https://pharos.nih.gov/targets).

### Pathway correlation analysis

The 50 hallmark pathways’ activities were figured using the Gene Set Variation Analysis (GSVA) by subjecting the gene sets acquired from the public website - MsigDB database (https://www.gsea-msigdb.org/gsea/index.jsp) to the GSVA R package. Then, we calculated the correlation between activity score and gene expression using the Pearson method.

### Construction of PRERGs risk signature

Three machine learning algorithms, including GBM, Coxboost, and Boruta, were performed to select potentially essential genes. Then, the dataset was separated into training and testing cohorts in terms of a ratio of 6:4. The training cohort was used to conduct Lasso-penalised Cox regression analyses based on 10-fold cross-validation from glmnet R-package. Genes were finally identified, and risk scores were calculated using the following equation:


$$\text{Risk score}=\text{sum}\;{\text{(coef}}_{\text{gene}}\times {\text{Expression}}_{\text{gene}}\text{)}$$

The receiver operating characteristic (ROC) curve was applied to estimate the predictive efficacy of the risk score. Then, the training cohort was divided into high- and low-risk groups based on the median risk score value. Kaplan-Meier survival curves were depicted to determine the prognostic difference between high- and low-risk groups. The same analyses were conducted in the testing and two external validation cohorts (KIRP and E-MTAB-1980 datasets).

### Clinical nomogram model

In multivariate Cox regression analysis, the risk score was adjusted by age, gender, stage, and grade. Then, based on the stepwise methods, the final variables were screened and utilized to construct a nomogram model to predict 3-/5-/7-year overall survival probability. Time-dependent ROC curve, calibration curve, and decision curve analysis were utilized to estimate the predicted efficacy and accuracy of the nomogram model.

### PPI network analysis and co-expression analysis

The protein-protein interaction analysis was performed by the public website (https://string-db.org/). The clinical-actionable genes were downloaded from previous research [22]. The correlation between clinical-actionable genes and PRERGs was estimated by Pearson methods in light of their mRNA expression, and the correlation was then shown through a network diagram.

### Pathway enrichment analysis

Gene ontology (GO), including cell component (CC), biological progression (BP), and molecular function (MF), were analyzed using the clusterProfiler R package. Gene set enrichment analysis (GSEA) of hallmark pathways was performed using GSEA software.

### Tumour immune infiltration analysis

Based on RNA-seq data from TCGA specimens, the CIBERSORT algorithm was used to quantify the proportion and distribution of tumour-infiltrating immune cells (TIICs) by presenting expression matrix and reference gene sets. We used the xCell R package to assess each sample’s immune, stromal, and tumour microenvironment scores. The gene sets of immune cells were downloaded from previous research [23]. Then, the single sample gene set enrichment analysis (ssGSEA) method was used to calculate the activities of immune cells.

### Statistical analysis

The unpaired t-test was used to compare the differences between the two groups. The association between variables and overall survival (OS) was assessed using univariate and multivariate Cox regression analyses. The survival probability differences of the two groups in Kaplan-Meier were assessed using the log-rank test. Pearson correlation coefficient (PCC) was used to quantify the correlation between the two groups. |PCC| >0.3 and *p*-value < 0.05 were regarded as significant.

### Data availability

All datasets generated for our research are introduced in the article.

## RESULTS

### Exosome-related genes identification

**Figure 1:**
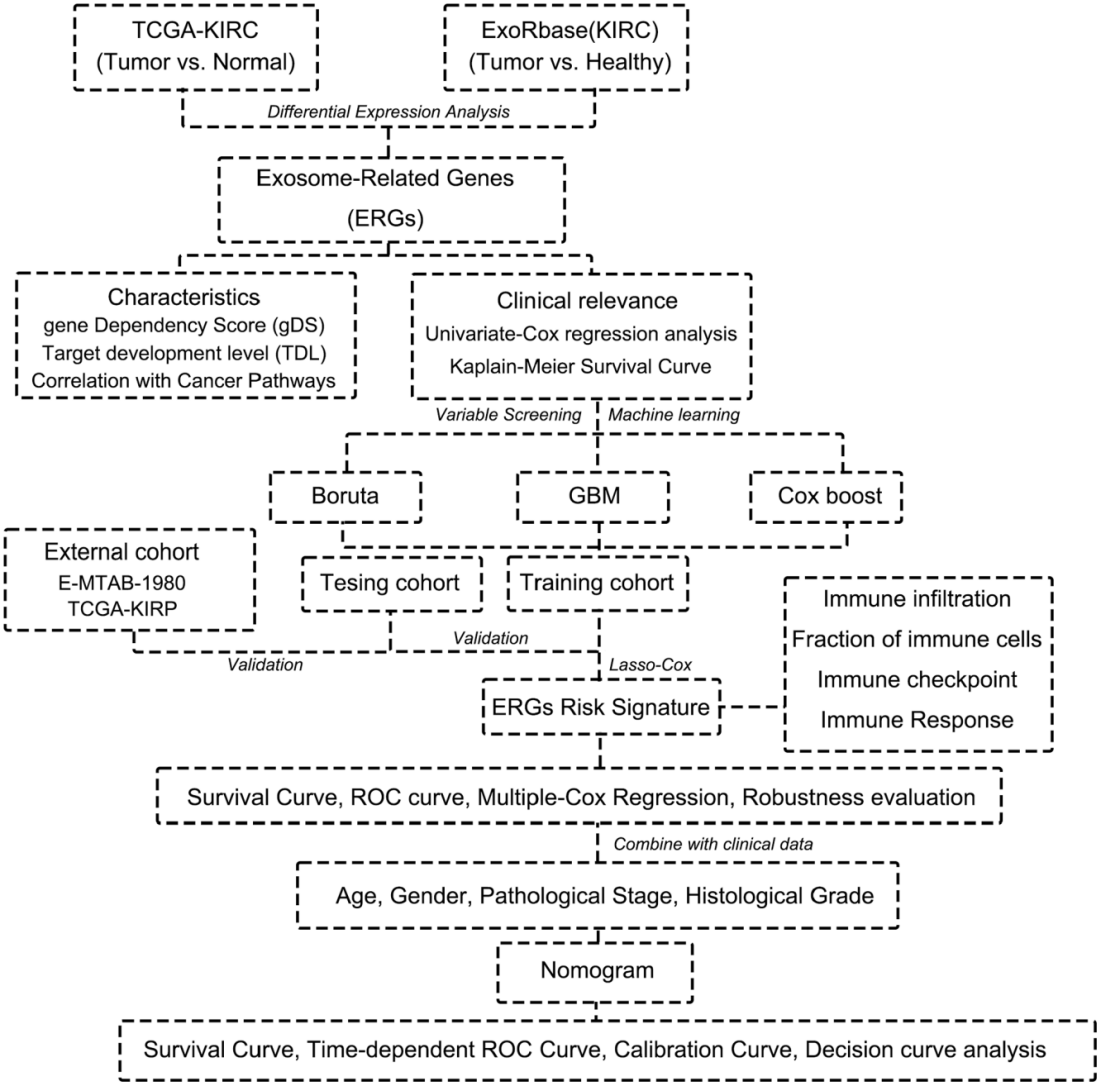
Flow chart of this research.

**Figure 2:**
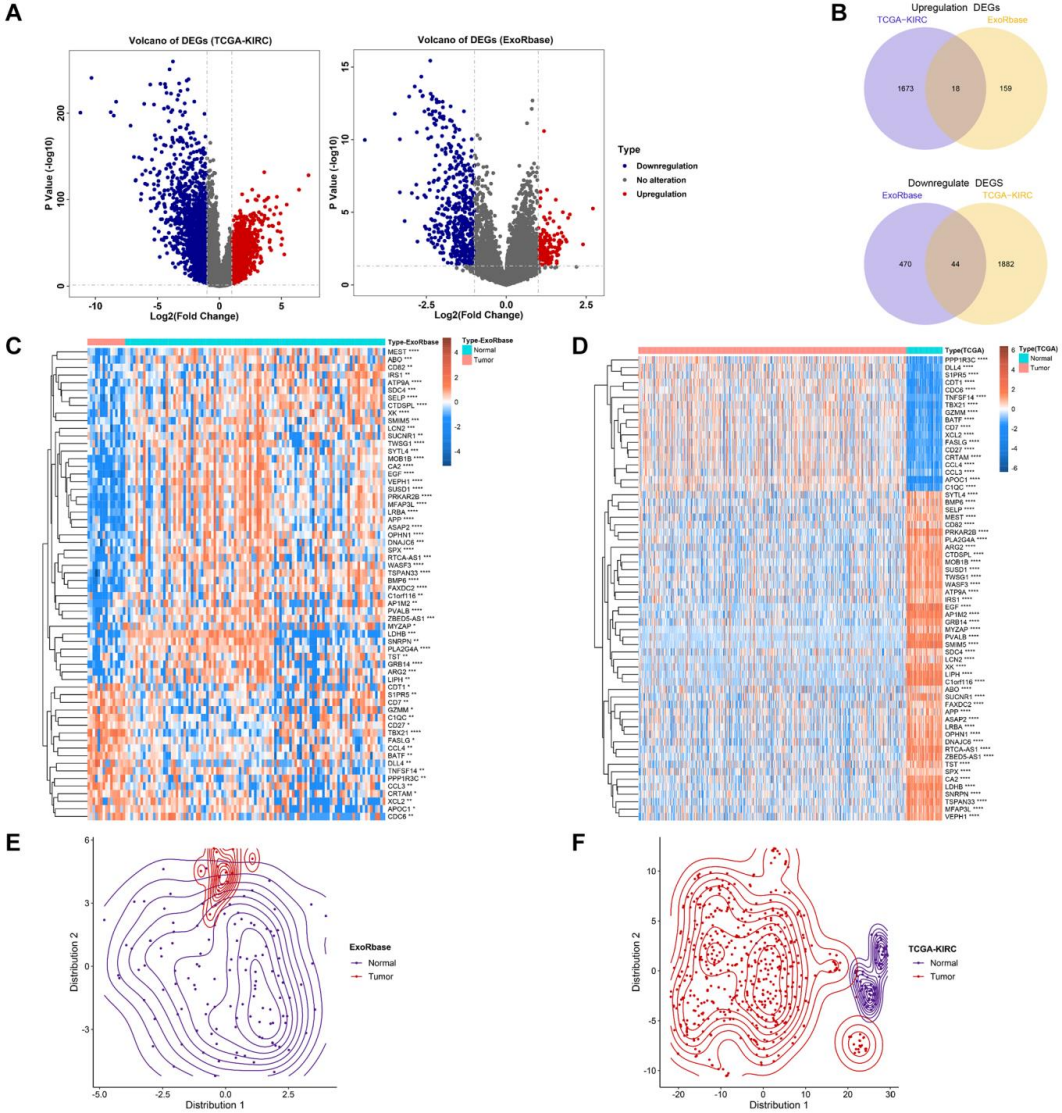
**Identification of exosome-related genes in KIRC.** (**A**) The volcano figure shows the differentially expressed genes between KIRC and normal control in exoRBase and TCGA-KIRC datasets. (**B**) The Venn diagram shows the common up-regulated genes and down-regulated genes in exoRBase and TCGA-KIRC datasets. (**C**, **D**) Heatmaps show the expression levels of ERGs in tumours and normal tissues. (**E**, **F**) T-SNE analyses were performed to show the distribution of tumour tissues and normal tissues based on ERG expression.

The Flow diagram is shown in Figure 1. We first conducted the differential expression analysis between KIRC and normal samples from TCGA and exoRBase. 3617 differential expression genes (DEGs) in TCGA-KIRC and 723 DEGs in exoRBase were eventually identified (Figure 2A). We further determined 18 common up-regulated genes and 44 common down-regulated genes in both datasets based on the Venn diagrams; these genes were named exosome-related genes (ERGs) (Figure 2B). The heatmaps and t-SNE analysis revealed that the mRNA expression of these ERGs could significantly distinguish normal and tumour samples (Figure 2C–2F). Therefore, we included these genes for further exploration.

### Clinical importance evaluation of ERGs

**Figure 3:**
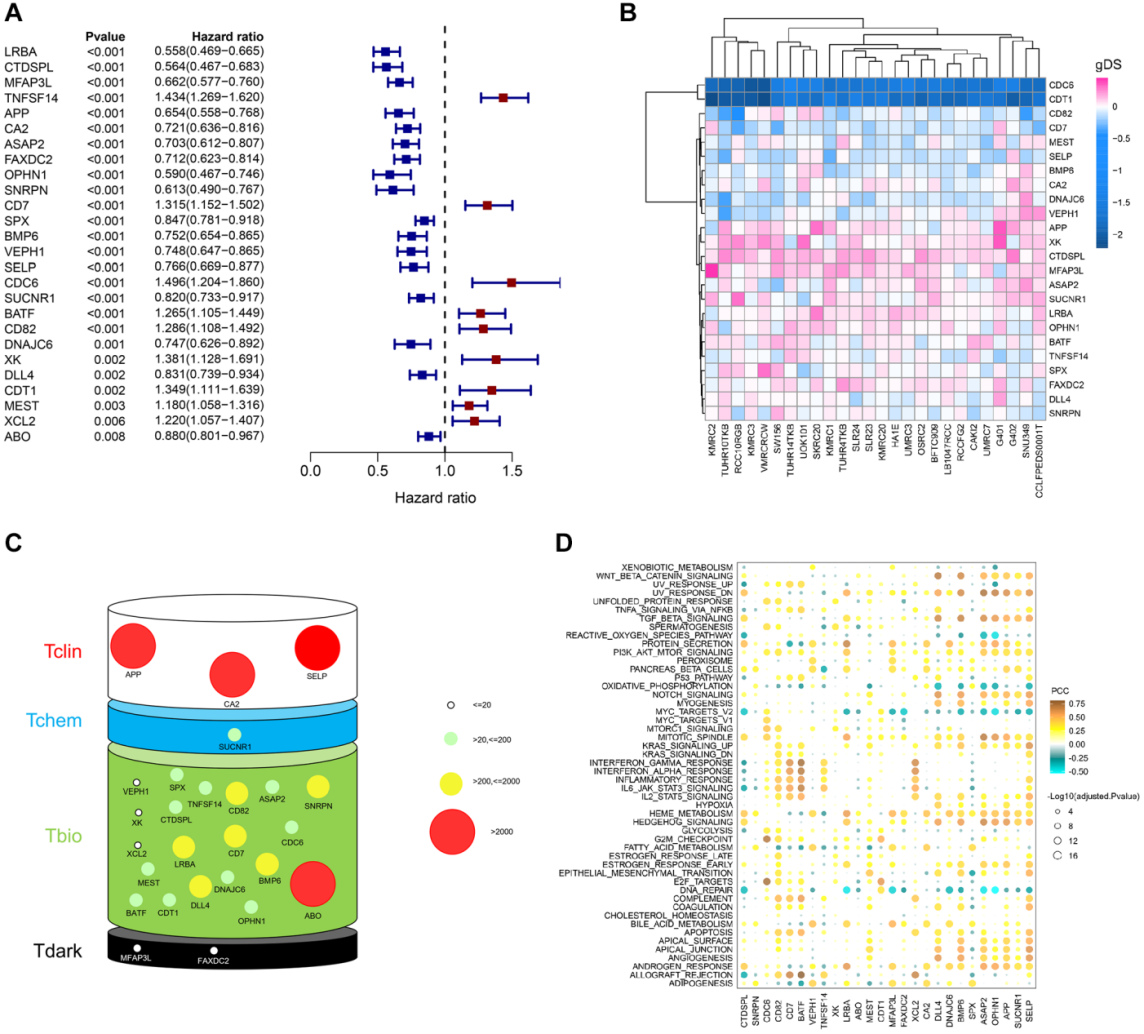
**Importance evaluation of PRERGs in KIRC.** (**A**) The forest diagram shows prognosis-related ERGs (PRERGs). (**B**) gDS score map shows the importance of a single gene to kidney cancer cells. (**C**) PubTator score and target development level (TDL) of PRERGs. The size of the circle represents different PubTator scores; the colour of the circle represents different TDLs. (**D**) The heatmap reveals a correlation between 50 cancer-related hallmark pathways activities and expression of PRERGs in KIRC.

Next, we performed a univariate Cox regression analysis to verify the relationship between prognosis and ERGs. 26 prognosis-related ERGs (PRERGs) were finally determined. Among these, 9 PRERGs were recognized as risk factors (TNFSF14, CD7, CDC6, BATF, CD82, XK, MEST, XCL2, and CDT1) while others as protective factors (LRBA, CTDSPL, OPHN1, SNRPN, APP, MFAP3L, ASAP2, FAXDC2, CA2, DNAJC6, VEPH1, BMP6, SELP, SUCNR1, DLL4, SPX, and ABO) (Figure 3A). The patients with high expression of risk genes showed a worse survival probability than those with low expression. In the same way, patients with low expression of protective genes showed a poorer prognosis than those with high expression (Supplementary Figure 1). Then, we used the gene dependency score (gDS) to assess the “importance” of 26 PRERGs. Several genes showed a low gDS in most kidney cancer cell lines, such as CD82, CD7, MEST, SELP, BMP6, CA2, DNAJC6, and VEPH1. CDC6 and CDT1, especially, represented the lowest gDS in kidney cancer cells, indicating that deletion of these two genes severely affects tumour cell viability (Figure 3B). In addition, we reviewed the target development levels (TDLs) and PubTator scores of these genes. We found APP, CA2, SELP, and ABO possess a higher PubTator score which is over 2000. PubTator scores of DLL4, CD7, LRBA, CD82, BMP6, and SNRPN are 200-2000, while others are lower than 200. Next, we analyzed the drug development status of PRERGs and their target development levels (TDLs). The TDLs were evaluated by Illuminating the Druggable Genome Knowledge Management Center, NIH. Three genes, APP, CA2, and SELP, have been developed targeted drugs, and SUCNR1 has been developed Tchem. Most genes do not have known approved drugs, and their small molecule activities do not satisfy the cutoffs of Tclin and Tchem. However, they are annotated with experimental evidence or gene ontology and are defined as Tbio. MFAP3L and FAXDC2 have very few studies and are defined as Tdark (Figure 3C). We further explored the relationship between these genes and cancer-related pathways by calculating the Pearson correlation coefficient (PCC) between the mRNA expression value of PRERGs and the activity score of cancer-related pathways. These PRERGs showed distinct associations with various pathways. CDC6 and CDT1, for instance, positively correlated with various cycle- and proliferation-related pathways, such as E2F targets and G2M checkpoint, indicating that they regulate cancer development by activating these pathways (Figure 3D). CD7, MEST, and XCL2 are highly correlated with IL6/JAK/STAT3, inflammatory response, and interferon-gamma/alpha response, revealing their peculiar regulation mechanism. Notably, CDC6 and CDT1 showed lower gDS and PubTator scores, are referred to as Tbio in Target development levels, and positively correlated with cancer-related pathways, which is worthy of further experimental exploration in RCC.

### PRERGs risk signature construction based on machine learning

**Figure 4:**
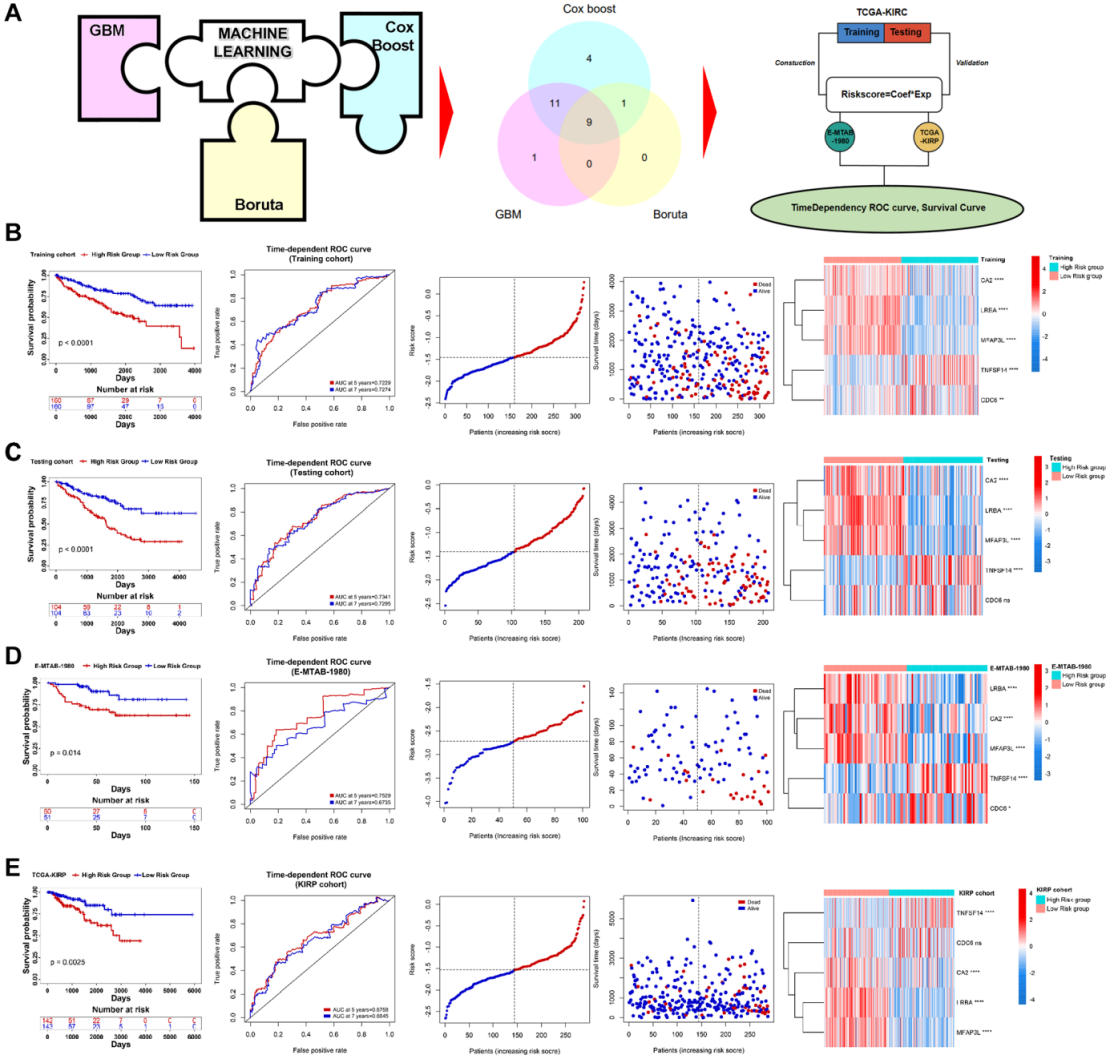
**Construction of PRERGs signature.** (**A**) The flow chart shows the machine-learning process. (**B**–**E**) Kaplan-Meier survival curve shows the survival differences in high- and low-risk groups. ROC curve shows the predicted accuracy of the risk score on the prognosis of the patients. The risk dot plot shows the number of dead and alive patients with gradually increasing risk scores. Heatmap shows the expression of 5 PRERGs that constructed the risk signature in the high- or low-risk group.

To explore the relationship between PRERGs and the prognosis of RCC patients, we conducted further screening by utilizing three machine learning methods, including GBM, Boruta, and Cox-Boost. 9 PRERGs were finally determined (Figure 4A). Then, we divided the TCGA-KIRC dataset into training and testing cohorts and conducted a Lasso-cox regression analysis based on the mRNA expression of these nine genes in the training cohort (Supplementary Figure 2A, 2B). A 5-gene risk signature (PRERGs risk signature) was finally determined, including TNFSF14, CA2, CDC6, LRBA, and MFAP43L. Based on their lasso coefficients and mRNA expression values, the risk score per patient was calculated by the formula as follows:


$$\begin{array}{l} \text{Risk score}=\text{TNFSF14}\times 0.1508474-\text{CA2}\times \\ \;0.01339085+\text{CDC6}\times 0.18011894-\text{LRBA}\times \\ \;0.31228396-\text{MFAP3L}\times 0.17567860 \end{array}$$

High-risk patients showed a poorer prognosis than low-risk patients (Figure 4B). The risk score showed a high accuracy in predicting the prognosis of KIRC patients (5 years ROC = 0.7229, 7 years ROC = 0.7274). In the high-risk group, the number of patients proved to be dead was far more than the number of patients in the low-risk group. In addition, we observed that CDC6 and TNFSF14 are highly expressed in the high-risk group, while CA2, LRBA, and MFAP3L are highly expressed in the low-risk group. Further validation was performed in the testing cohort, E-MTAB-1980, and KIRP datasets, and the results bear resemblance to the training cohort (For testing cohort: 5 years ROC = 0.7341, 7 years ROC = 0.7295; for E-MTAB-1980: 5 years ROC = 0.7529, 7 years ROC = 0.6735; for TCGA-KIRP: 5 years ROC = 0.6758, 7 years ROC = 0.6645) (Figure 4C–4E). In addition, we found that these genes interacted with different proteins and positively or negatively correlated with different clinically actionable genes, indicating that they might regulate cancer by different mechanisms and interact with different substrates (Supplementary Figure 3A, 3B).

### PRERGs risk signature correlated with clinical information

**Figure 5:**
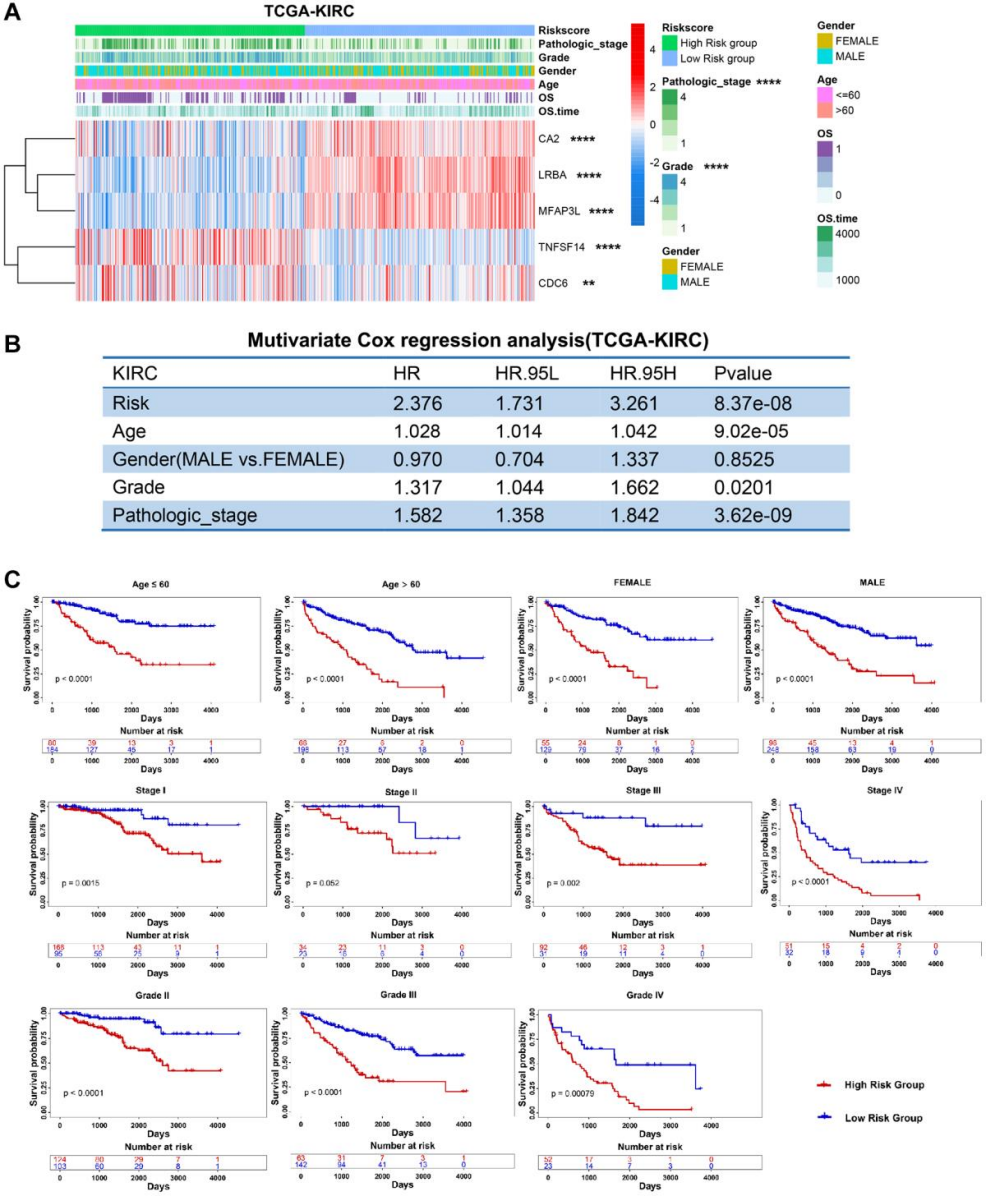
**Clinical relevance and robustness of PRERGs risk score in TCGA-KIRC dataset.** (**A**) The heatmap shows the distribution of clinical variables in the high- or low-risk groups. (**B**) The results of multiple Cox-regression analysis in the TCGA-KIRC dataset. (**C**) Kaplan-Meier shows the prognostic differences between high- and low-risk groups in classified clinical variables. ^*^P-value < 0.05; ^**^P-value < 0.01; ^***^P-value < 0.001; ^****^P-value < 0.0001.

Next, we excavated the relationship between clinical status and risk score in three RCC datasets. We found that PRERG’s risk score was associated with pathological stage and grade of TCGA-KIRC. Specifically, patients with advanced stages and grades are mainly distributed in the high-risk group (Figure 5A). The risk score was then adjusted by other clinical variables such as age, gender, grade, and stage during multivariate Cox regression analysis. The results showed that the PRERG risk score was an independent prognostic factor (Figure 5B). What is more, the risk scores were practical in different clinical subgroups. Patients with high risk always showed worse outcomes than patients in low-risk groups (Figure 5C). The same analyses were performed in another two datasets and revealed similar results (Supplementary Figures 4 and 5). Notably, the risk score showed no correlation with stage and grade in the E-MTAB-1980 dataset, which might be due to small sums of samples. However, we still observed that more patients with advanced stages and grades were distributed in the high-risk group than in the low-risk group. Despite different datasets, PRERGs still acted as an independent prognostic factor, and the high-risk group invariably showed a lower survival rate than the low-risk group, indicating that PRERGs were steady and robust in predicting the prognosis of RCC patients.

### Clinical model construction

**Figure 6:**
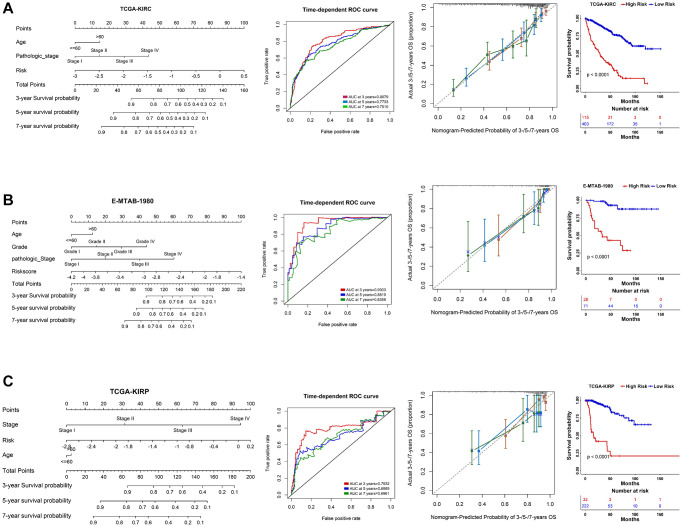
**Nomogram construction in three RCC datasets.** (**A**–**C**) A nomogram was constructed based on PRERGs and clinical variables to predict the 3-/5-/7-year survival probability of the patients in the TCGA-KIRC, TCGA-KIRP, and E-MTAB-1980 datasets. ROC and calibration analysis were performed to estimate the predicted accuracy. The Kaplan-Meier survival curve was depicted to show the prognosis difference of high- or low-risk groups which was calculated by nomogram.

Using multivariate Cox regression analysis and stepwise regression method in the TCGA-KIRC dataset, a nomogram model was subsequently established to estimate 3-/5-/7-year survival probability (Figure 6A). Time-dependent ROC and calibration curves revealed that the model possesses a discriminative accuracy (3 years AUC = 0.8079; 5 years AUC = 0.7733; 7 years AUC = 0.7510). Decision curve analysis (DCA) illustrates that this nomogram model could receive more clinical benefit than single variables (Supplementary Figure 6A). Based on the nomogram model, a risk score was finally determined, and the patients in different risk groups showed significant survival differences (Figure 6A). The same analyses were performed in another two external validation cohorts, and the results proved to be the same trends (Figure 6B, 6C; Supplementary Figure 6B, 6C). We then compared our PRERG signature with nine other risk signatures [24–32]. PRERGs risk signature showed the top 3 of AUC for 3-/5-/7-year prognosis of the KIRC patients (Supplementary Figure 7A–7C), indicating that our risk signature has a higher predicted accuracy.

### Potential molecular mechanism

To demonstrate the potential molecular mechanism between the high PRERGs risk group and the low PRERGs risk group, we performed enrichment analysis by submitting the differentially expressed genes screened from the high-/low-risk group to the clusterProfiler R package. We found cell components, such as blood microparticles, immunoglobulin complex, immunoglobulin complex circulating, plasma lipoprotein particle, and protein-lipid complex, highly enriched in the high-risk group. In the case of biological progression, antibacterial humoral response, antimicrobial humoral response, positive regulation of T cell migration, regulation of CD4-positive alpha-beta T cell differentiation, and regulation of T cell migration are highly enriched in the high-risk group. Meanwhile, molecular function, antigen binding, chemokine activity, chemokine receptor binding, fatty acid binding, and immunoglobulin receptor binding are highly enriched in high-risk groups (Supplementary Figure 8A–8C). By presenting the mRNA expression profile ordered by risk score per sample to GSEA software, we found no hallmark pathways significantly enriched in a high-risk group. In contrast, several pathways showed a correlation with the low-risk group. The top 5 hallmark pathways that were enriched in the low-risk group are adipogenesis, heme metabolism, fatty acid metabolism, bile acid metabolism, and peroxisome pathways, indicating that low enrichment of these genes in the high-risk group might be the reason causing poor outcomes in the patients (Supplementary Figure 8D).

### Immune infiltration and therapeutic response

**Figure 7:**
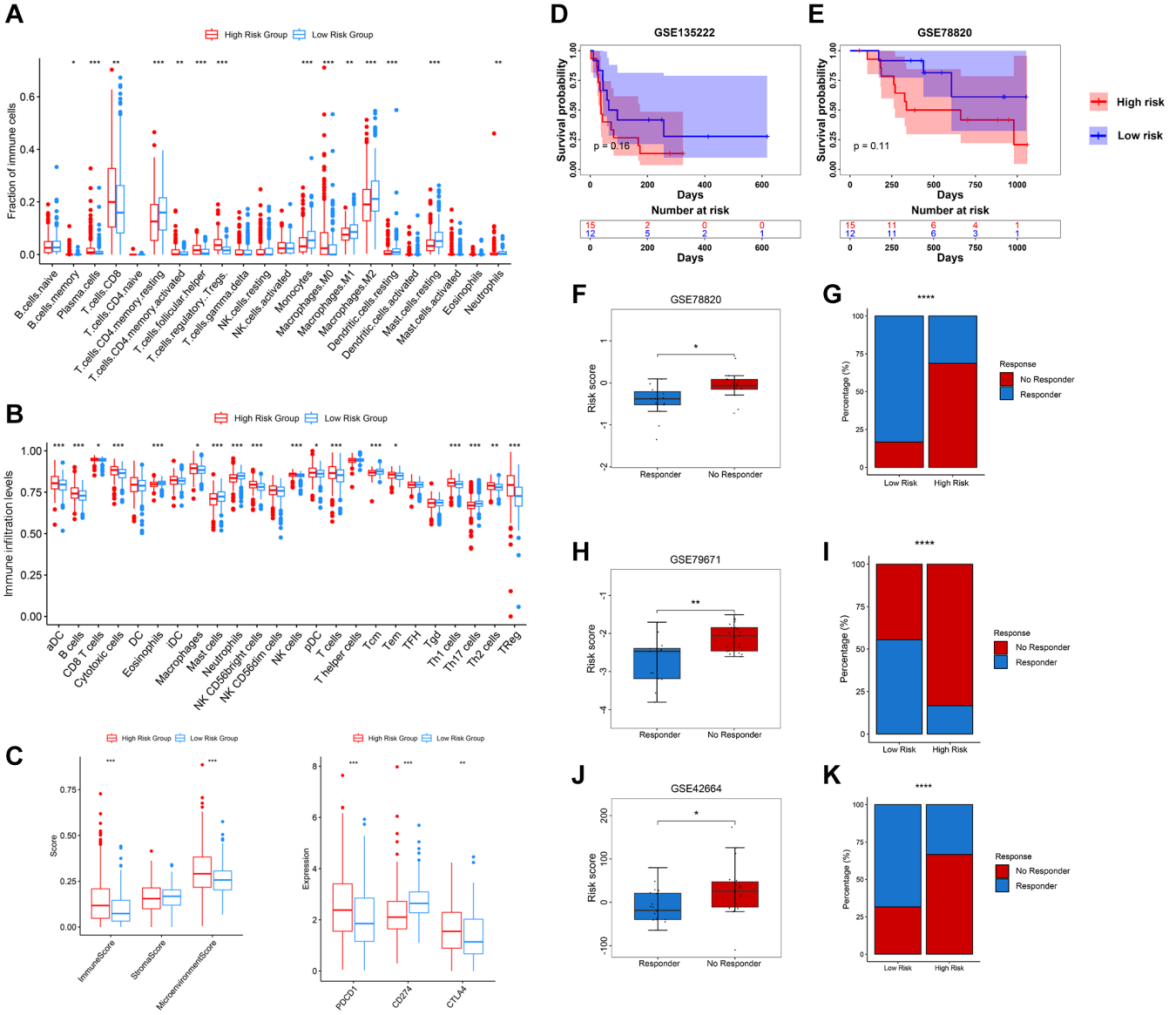
**Relevance between tumour immune infiltration and PRERGs risk signature.** (**A**) Fraction of immune-related cells in the high- or low-risk group. (**B**) Activity score of immune-related cells in the high- or low-risk group. (**C**) Immune score, stromal score, microenvironment score, and three immune checkpoint expressions in the high- or low-risk group. (**D**, **E**) Kaplan-Meier survival curves were portrayed to show the survival difference in the high- or low-risk group in the GSE135222 and GSE78820 datasets. (**F**, **G**) Boxplot shows the risk scores of CR/PR and SD/PD groups in the GSE78820 dataset. The barplot shows the distribution of high- or low-risk groups in CR/PR and SD/PD groups. (**H**, **I**) Boxplot shows the risk score of the response and no-response groups in the GSE79671 dataset. The barplot shows the distribution of high- or low-risk groups in the response and no-response groups. (**J**, **K**) Boxplot shows the risk score of CR/PR and SD/PD groups in the GSE42664 dataset. The barplot shows the distribution of high- or low-risk groups in CR/PR and SD/PD groups. Abbreviations: CR: complete response; PR: partial response; SD: stable disease; PD: progressive disease. ^*^P-value < 0.05; ^**^P-value < 0.01; ^***^P-value < 0.001; ^****^P-value < 0.0001.

We further explored the infiltration levels of different immune cells in these two groups. As expected, we found that CD4+ T cells and CD8+ T cells show higher fraction and infiltration levels in high-risk groups. However, we observed that Tregs are highly enriched in the high-risk group (Figure 7A, 7B). Furthermore, the immune and microenvironment scores in the high-risk group show a high enrichment, while the stroma score shows no significant difference in these two risk groups (Figure 7C). The immune checkpoints PDCD1 (also known as PD1) and CTLA4 show a higher expression in the high-risk group, while CD274 (also known as PDL1) shows a higher expression in the low-risk group (Figure 7C). To explore whether the PRERGs risk signature could predict immune therapy response, we further carried out Tumor Immune Dysfunction and Exclusion (TIDE) (http://tide.dfci.harvard.edu/) analysis by presenting a normalized gene expression profile of TCGA-KIRC [33]. The results reveal that most responders (R) harboured in low-risk groups, though the distribution did not show statistical significance (Supplementary Figure 9). We then validated the speculation using four other external immune therapy cohorts (including GSE135222, GSE78820, GSE79671, and GSE42664). We extracted the mRNA expression of PRERGs from normalized datasets and then calculated the risk score. Then, we matched the risk score with the clinical data of each dataset; among these, GSE135222 and GSE78820 contain survival data that enable us to perform survival analysis. The high- and low-risk groups were determined by ideal cutoff value, and we also observed that high-risk group patients showed low survival probability in these two cohorts, even though the difference was not significant statistically, that might be due to the small sums of samples (Figure 7D, 7E). We further analyzed another three immune therapeutic cohorts (GSE78820, GSE79671, and GSE42664); conformably, no responders in these datasets showed a higher risk score, while those proved to be responders represented a lower risk score (Figure 7F, 7H and 7J). We calculated the proportion of responders and non-responders harboured in the high- or low-risk group. The results revealed that a higher proportion of no-responders were distributed in the high-risk group. In contrast, only a slight collection of non-responders was located in the low-risk group (Figure 7F–7K). These results suggested that the PRERG risk signature might act as a predictor for predicting the reaction of patients who received immune therapy.

## DISCUSSION

Kidney cancer is one of the common malignant tumours of the urinary system. Its early symptoms are hidden and difficult to detect. Metastasis usually occurs after the appearance of clinical symptoms [34]. Its incidence rate is increasing, and the incidence rate in developed countries is generally higher than in developing countries [2]. One of the primary reasons for the increase in incidence is the improvement of medical resources and the public’s awareness of their health [35]. Although early diagnosis and treatment have reduced the mortality rate of renal cancer, metastasis after treatment still exists, so there is a need to find a new therapeutic target to treat this type of disease effectively.

Recent research has revealed that exosomes, as part of the extracellular environment, can remodel the extracellular environment and transmit signals and molecules to neighbouring cells [12]. Tumour-derived exosomes can secrete miRNA to induce tumour cell metastasis and mediate the reconstruction of the tumour microenvironment [36, 37]. In addition, exosomes are critical for pre-metastatic niche (PMN) formation and metastasis by intercellular communication between tumour cells and the distant organ microenvironment [13]. Several studies demonstrated that tumour-derived exosomes promote the development of renal cancer cells and are non-invasive bioindicators for clinical diagnosis and assessment of renal cancer prognosis [19, 38]. Our study identified 26 potential exosome-related genes based on exoRBase-KIRC and TCGA-KIRC datasets. Gene Dependency Score, Target development levels, and cancer pathway correlation analysis were performed to explore the potential function and clinical targets. We found several interesting genes, such as APP, which has been developed as Tclin. APP is overexpressed with APLP in multiple cancers, including glioblastoma and breast, pancreatic, lung, colon, and prostate cancer, which is known to participate in the progression, proliferation, and migration of cancer cells [39]. However, the univariate Cox regression result revealed that APP is a protective factor for the KIRC patient’s prognosis. gDS showed that APP deletion slightly affects several kidney cancer cells’ viability, which reduces the possibility of APP acting as a target in kidney cancer. CDC6 and CDT1, which act as risk factors for patients’ prognosis, showed a low enough gDS in all kidney cancer cells to show that their deletion severely influences cells’ viability. Even though little research concerns these two genes, there is still some evidence indicating that they will be novel therapeutic targets. For CDC6, evidence has been accumulated that it acts as an oncogene promoting tumour progression and as a potential driver of tumourigenesis [40]. Aberrant CDT1 expression has been reported to promote tumourigenesis, and its small molecular inhibitor showed an obvious tumour-inhibition function by inducing DNA damage [41]. In addition, we also found that these two genes show a highly positive correlation with multiple tumour-related pathways, such as E2F targets and G2M checkpoint pathways. Among these, E2F pathways have been found to promote cancer and are considered for developing therapeutic strategies [42]. Therefore, it is considered that CDC6 and CDT1 promote kidney cancer by regulating these oncogenic pathways. Recent research also found that down-regulated CDC6 expression in bladder cancer cells and exosomes could inhibit the malignant processes of bladder cancer cells [43], revealing that targeting this exosome-derived gene might be a promising treatment strategy against tumours. However, whether these exosome-derived genes act as promising targets in kidney cancer still needs further validation.

Previous research has pointed to the use of big clinical data to construct predictive models as an important strategy in current clinical practice [44, 45]. In this study, we used three machine learning methods to screen genes that potentially closely correlate with the prognosis of kidney cancer patients. Then, Lasso-cox regression was performed to determine the final PRERGs; 5 genes were selected to construct the PRERGs risk score. CDT1 was excluded after the screening process, whereas CDC6 was included. To some degree, CDC6 and CDT1 might act as similar functions, not only for their resemblance on gDS score but for their consistent correlation with potential cancer-related pathways. Considering that increasing CDC6 expression is largely correlated with poor prognosis of KIRC patients, it is reasonable to expel CDT1 during the progression of Lasso-cox regression. In multiple kidney cancer datasets, PRERGs risk scores were practical and acted as an independent prognostic factor, suggesting that PRERGs risk score was a better predictive factor on patient prognosis.

Over the past decade, cancer immunotherapy, which eliminates tumour cells by modulating the patient’s immune system, has revolutionized the contours of cancer treatment [46]. Thus, the immune system and therapy are scientific issues of deep interest in current clinical cancer practice. In our study, we found multiple immune-related progressions highly enriched in the high-risk group, indicating that high-risk patients showed an activation of the immune system. Apparently, immune activation is largely considered good for patients’ prognosis, but high-risk patients still represent poor clinical outcomes. By relating to the immune infiltration microenvironment, we found that CD8+ T-cells showed a higher proportion and activity in the high-risk group, while CD4+ T-cells naive and memory resting CD4+ T-cells were adverse. In addition, we also observed that Treg cells were highly enriched in the high-risk group. Tregs are reported to mediate the immune system and prevent autoimmune disease [47]. In addition, Tregs can inhibit effector T cell proliferation and cytokine production, thus limiting immune-mediated inflammation [47]. Due to their inhibition of effector T-cell responses, Tregs have a risky impact on cancer patients’ survival [48]. Therefore, we hypothesize that the high activity of Tregs might be the reason for the poor prognosis of the patients in the high-risk group. Notably, the survival and function of Tregs have been revealed to rely on lipid metabolism, and free fatty acid could match their metabolic requirement [49]. In our study, we found the molecular function of fatty acid binding highly enriched in the high-risk group; this might have provided a survival environment for Tregs in this cohort. Exosomes have been found to be released by cancer cells to alter the tumour microenvironment, such as altering stromal cell types to promote cancer progression and promote tumour angiogenesis [50]. In addition, exosomes can inhibit immune response by expressing molecules such as PD-L1 [51]. Tumour-derived exosomes can inactivate CD8+ T cells and promote regulatory T cell expression to suppress the immune system [52]. These findings revealed that exosome-based immunotherapy was a promising therapeutic strategy. In our research, we found that KIRC patients in the low-risk group might benefit from immune therapy through TIDE analysis. Interestingly, the results of three other immune therapy datasets bear a resemblance to the finding; that is, low-risk groups show a higher proportion of the immune response rate. Even though these immune therapy datasets come from different cancer cohorts, they also provide a potential verification that the PRERGs risk signature could stratify the specific patients who might benefit from immune therapy. After conducting immune therapy, low-risk patients might receive long-term survival, while high-risk patients who are treated with immune therapy should consider other treatment strategies as precautions. For example, considering TNFSF14 and CDC6 show higher expression in the high-risk group, they could be regarded as potential targets for treating RCC patients in the high-risk cohorts. However, whether these exosome-derived genes could act as potential targets and be applied to clinics still needs large-scale pre-clinical and clinical trials to verify.

## CONCLUSION

Our study constructed a PRERGs risk signature based on large-scale exosome-related and RCC tissue datasets. The risk signature could independently predict the prognosis of RCC patients and immunotherapeutic response. However, more research should be conducted to learn the concrete mechanism of these exosome or tissue-derived genes in regulating RCC. We hope these organized data can provide a theoretical basis for further experimental research on exosomes in KIRC.

## Supplementary Materials

Supplementary Figures
