# Supplementary material for: Construction of exosome-related genes risk model in kidney cell carcinoma predicts prognosis and immune therapy response
Source: Aging (Albany NY). 2024 May 7;16(9):7622–46. doi: 10.18632/aging.205767 (PMC11132023; doi:10.18632/aging.205767)
Supplement: Supplementary Figures [file aging-16-205767-s001.pdf]

SUPPLEMENTARY FIGURES

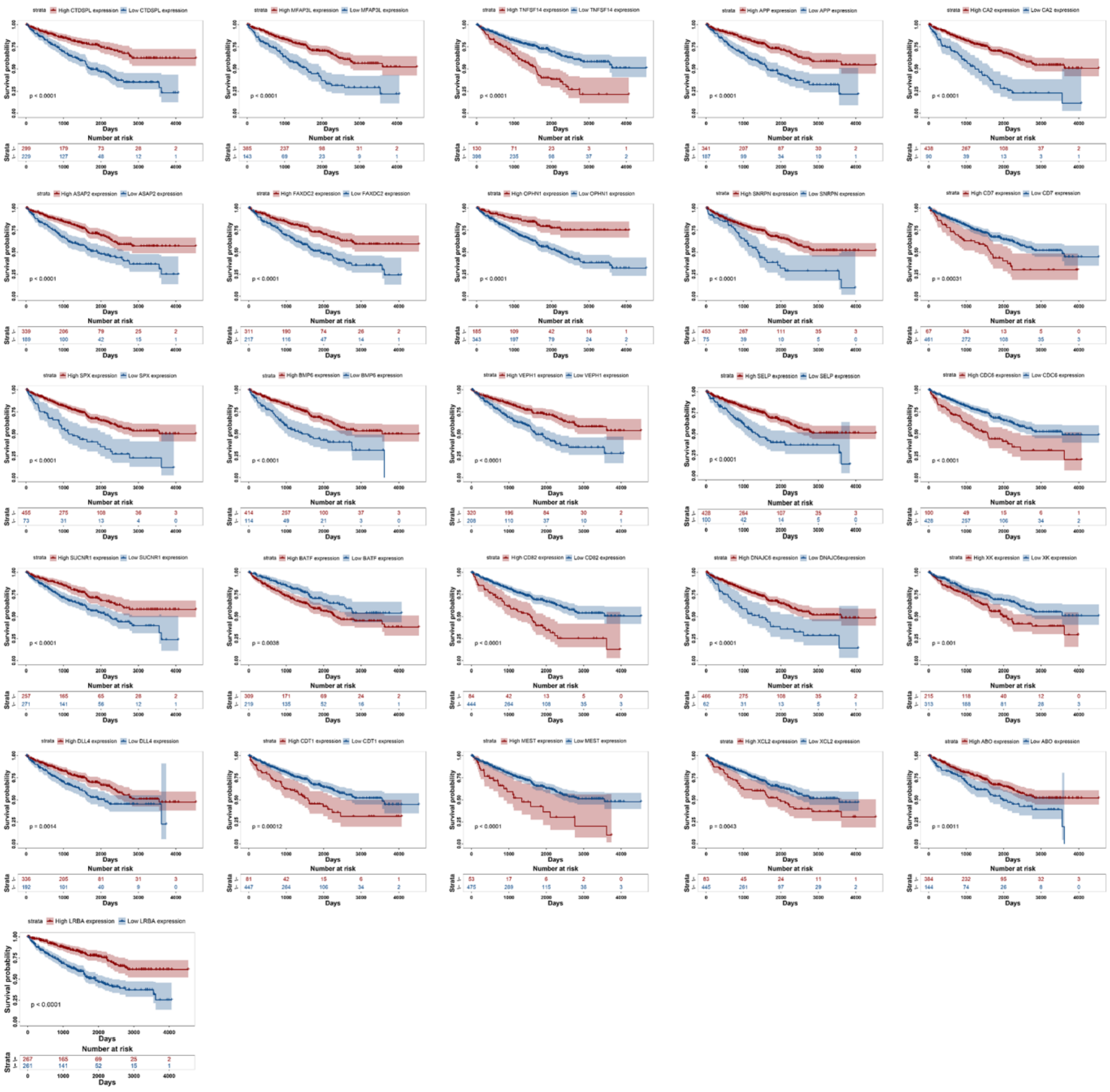

Supplementary Figure 1. The samples of TCGA-KIRC were separated into high/low expression groups according to the ideal cut-off value determined by the surv_cutpoint R function; the Kaplan-Meier survival curve was depicted to show the survival differences of the high/low expression groups.

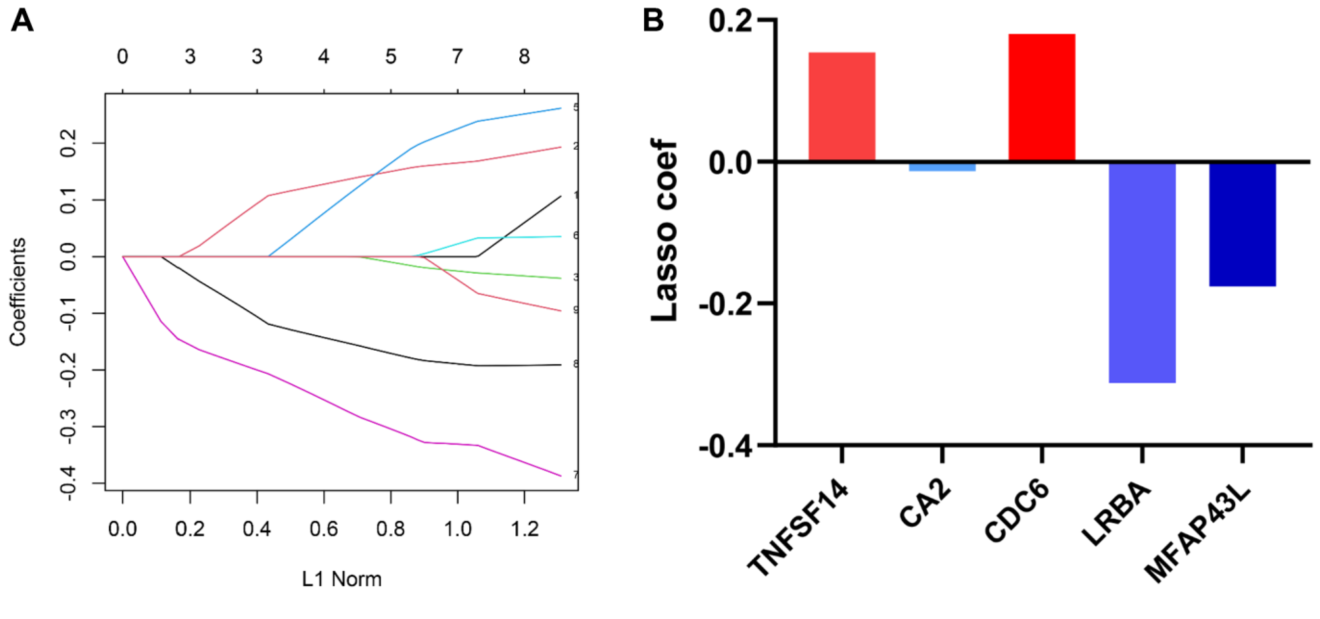

Supplementary Figure 2. The Lasso-cox regression progression (**A**) and the coefficients of ERGs (**B**).

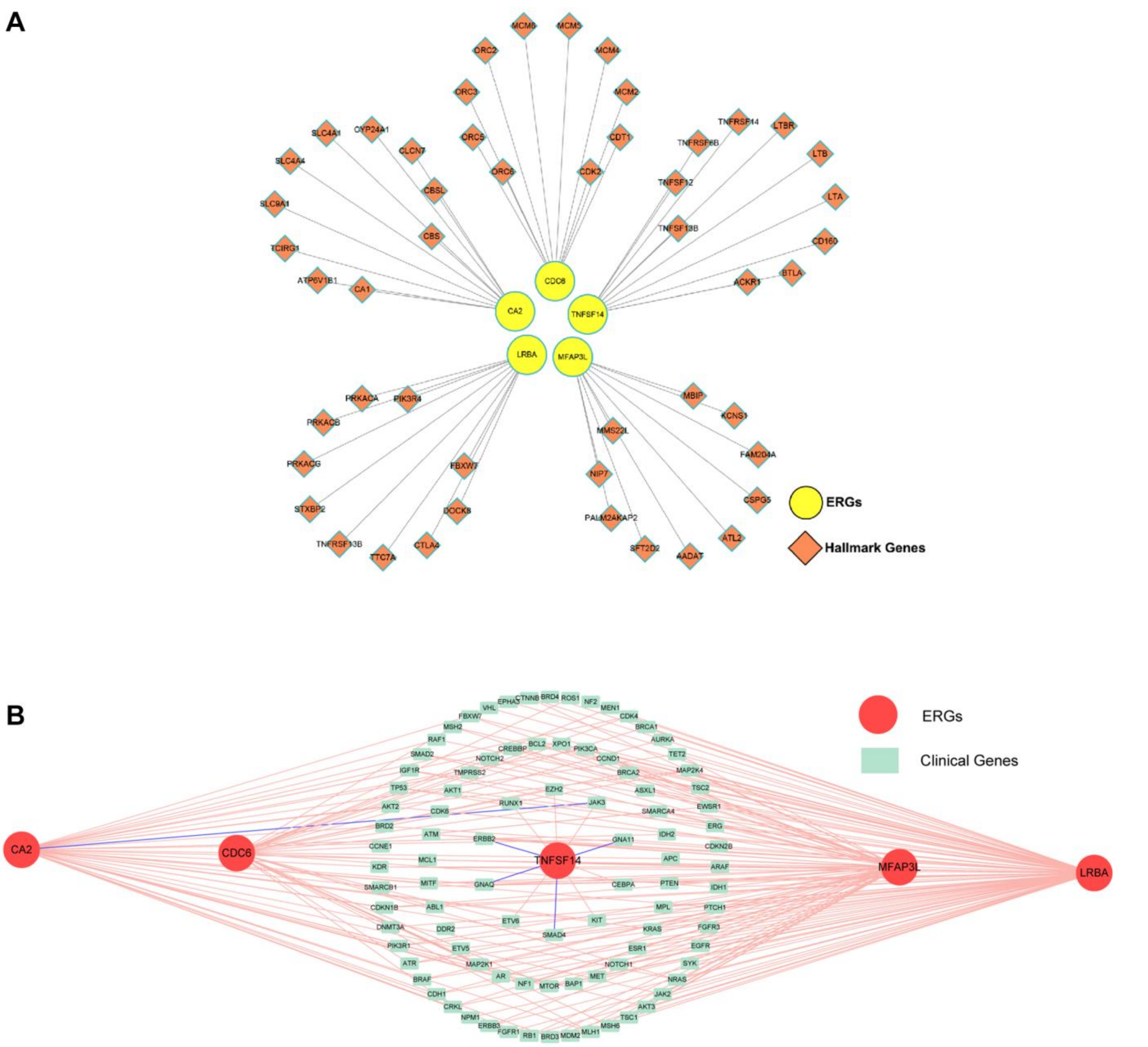

Supplementary Figure 3. (**A**) The network diagram reveals the potential mutually interacted proteins with the 5 PRERGs. (**B**) The network revealed the relation between 5 PRERGs and clinically actionable genes in mRNA expression levels.

A

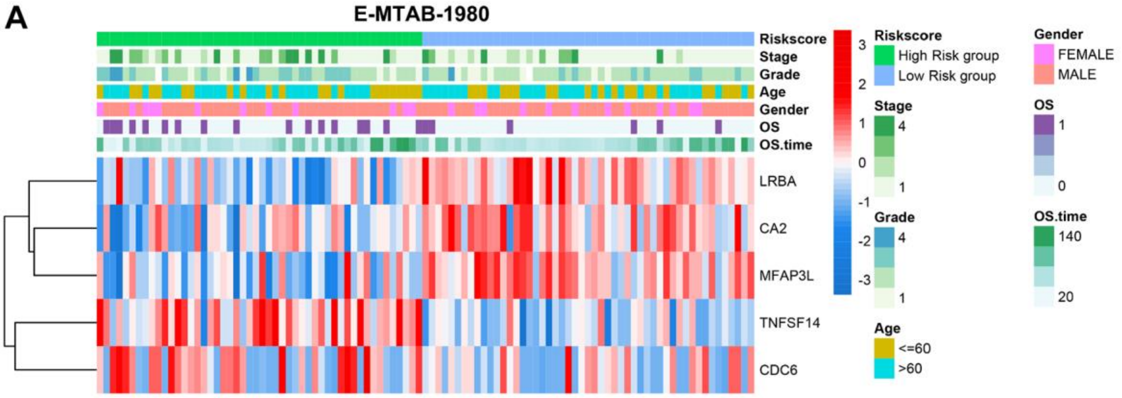

B

Mutivariate Cox regression analysis(E-MTAB-1980)

	HR	HR.95L	HR.95H	Pvalue
Risk	3.418	1.2628	9.251	0.0156
Age	1.014	0.9736	1.056	0.5010
Gender(MALE vs.FEMALE)	2.870	0.8038	10.246	0.1045
Grade	1.508	0.7932	2.865	0.2102
Pathologic_stage	2.274	1.5611	3.313	1.87e-05

C

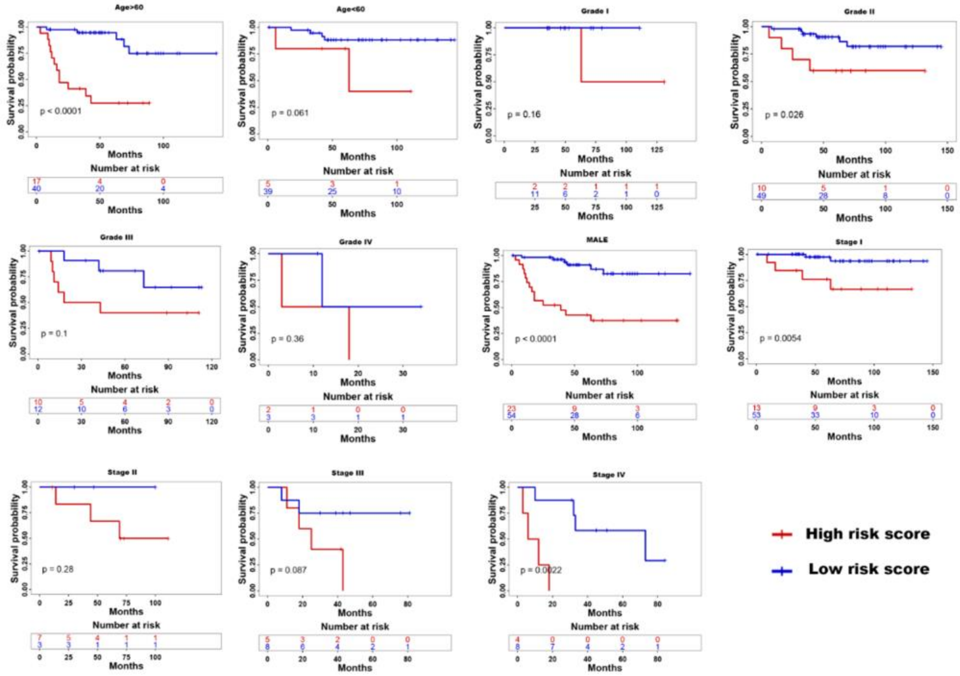

Supplementary Figure 4. Clinical relevance and robustness of PRERGs risk score in E-MTAB-1980 dataset. (**A**) The heatmap shows the distribution of clinical variables in the high- and low-risk groups. (**B**) Multiple Cox-regression analysis results in the E-MTAB-1980 dataset. (**C**) Kaplan-Meier shows the prognostic differences between high- and low-risk groups in classified clinical variables.

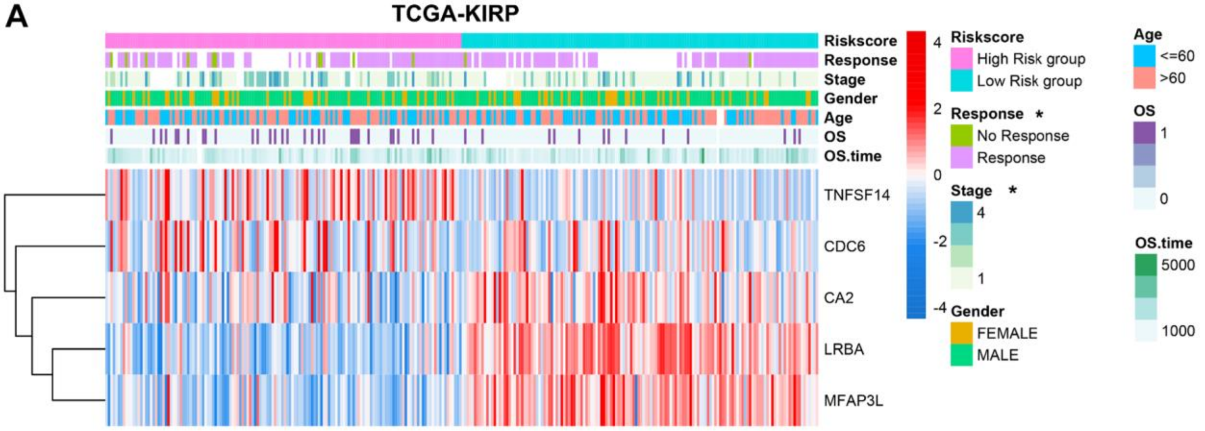

B

Mutivariate Cox regression analysis(TCGA-KIRP)

	HR	HR.95L	HR.95H	Pvalue
Risk	2.3785	1.1781	4.802	0.0156
Age	1.0038	0.9750	1.034	0.7980
Gender(MALE vs.FEMALE)	0.7686	0.3803	1.554	0.4636
Pathologic_stage	2.2714	1.6783	3.074	1.08e-07

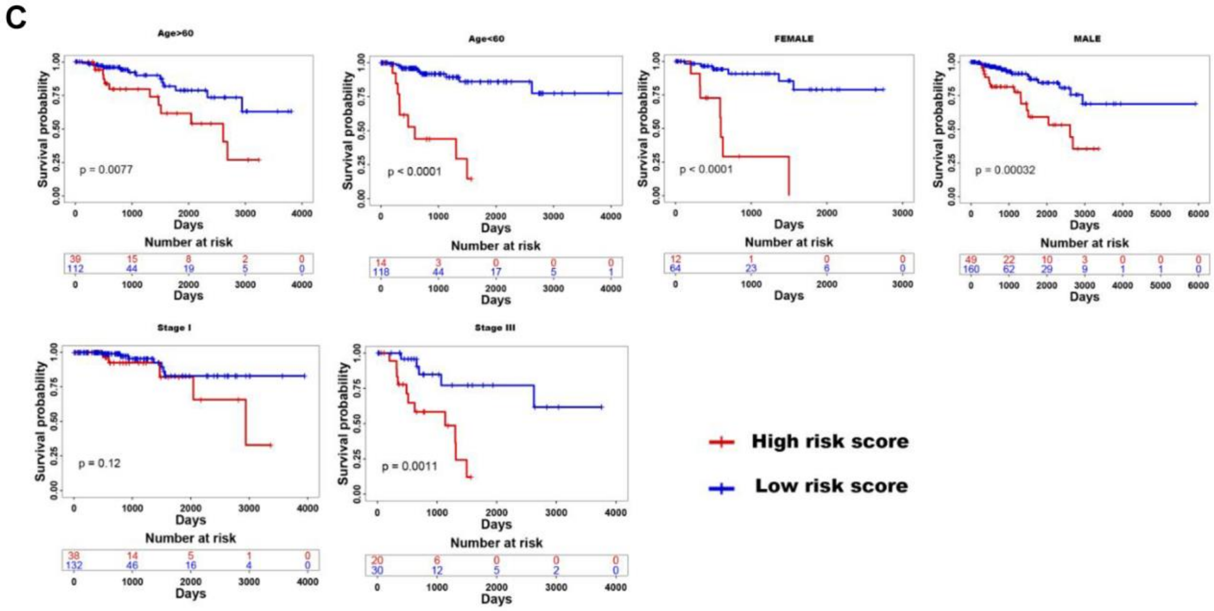

Supplementary Figure 5. Clinical relevance and robustness of PRERGs risk score in TCGA-KIRP dataset. (**A**) Heatmap shows the distribution of clinical variables in high- and low-risk groups. (**B**) Multiple Cox-regression analysis results in the TCGA-KIRP dataset. (**C**) Kaplan-Meier shows the prognostic differences between high- and low-risk groups in classified clinical variables.

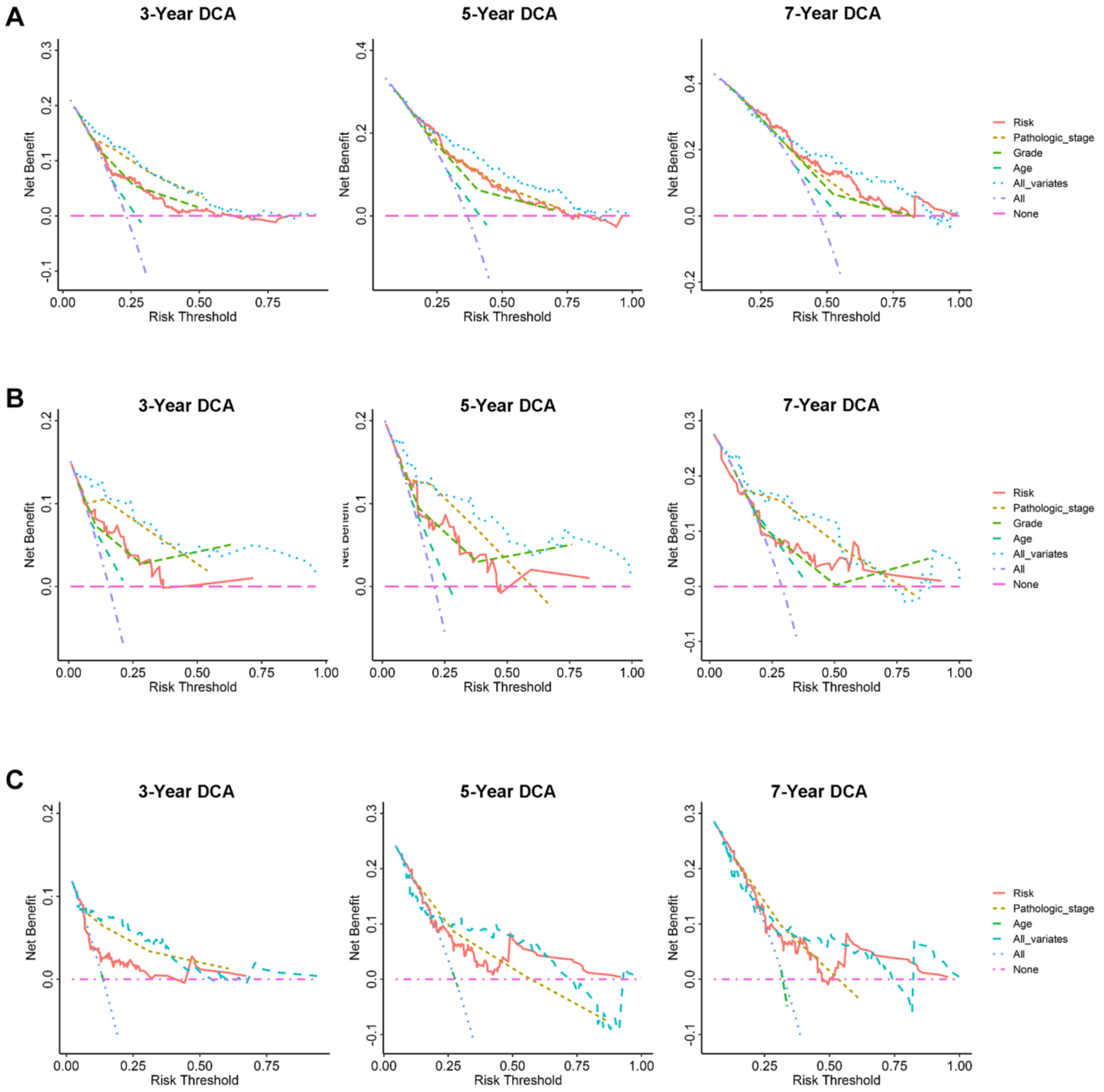

Supplementary Figure 6. Decision curve analysis was conducted to evaluate the clinical benefit of the constructed nomogram on predicting prognosis in TCGA-KIRC (**A**), E-MTAB-1980 (**B**), and TCGA-KIRP (**C**) datasets.

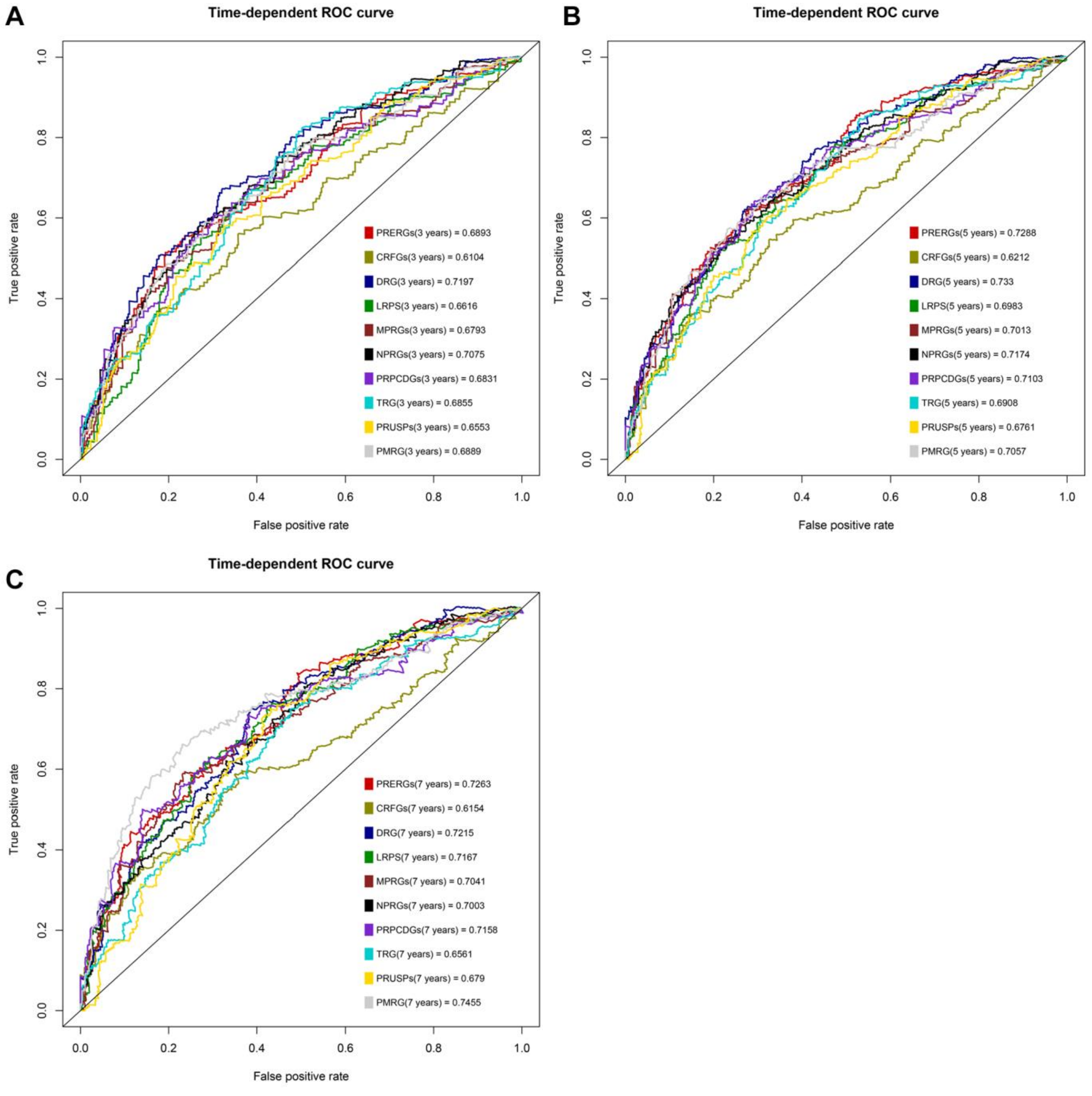

Supplementary Figure 7. (**A**–**C**) ROC curve was used to estimate the predicted accuracy of PRERGs risk signature and other existing risk signatures for 3-/5-/7-year survival.

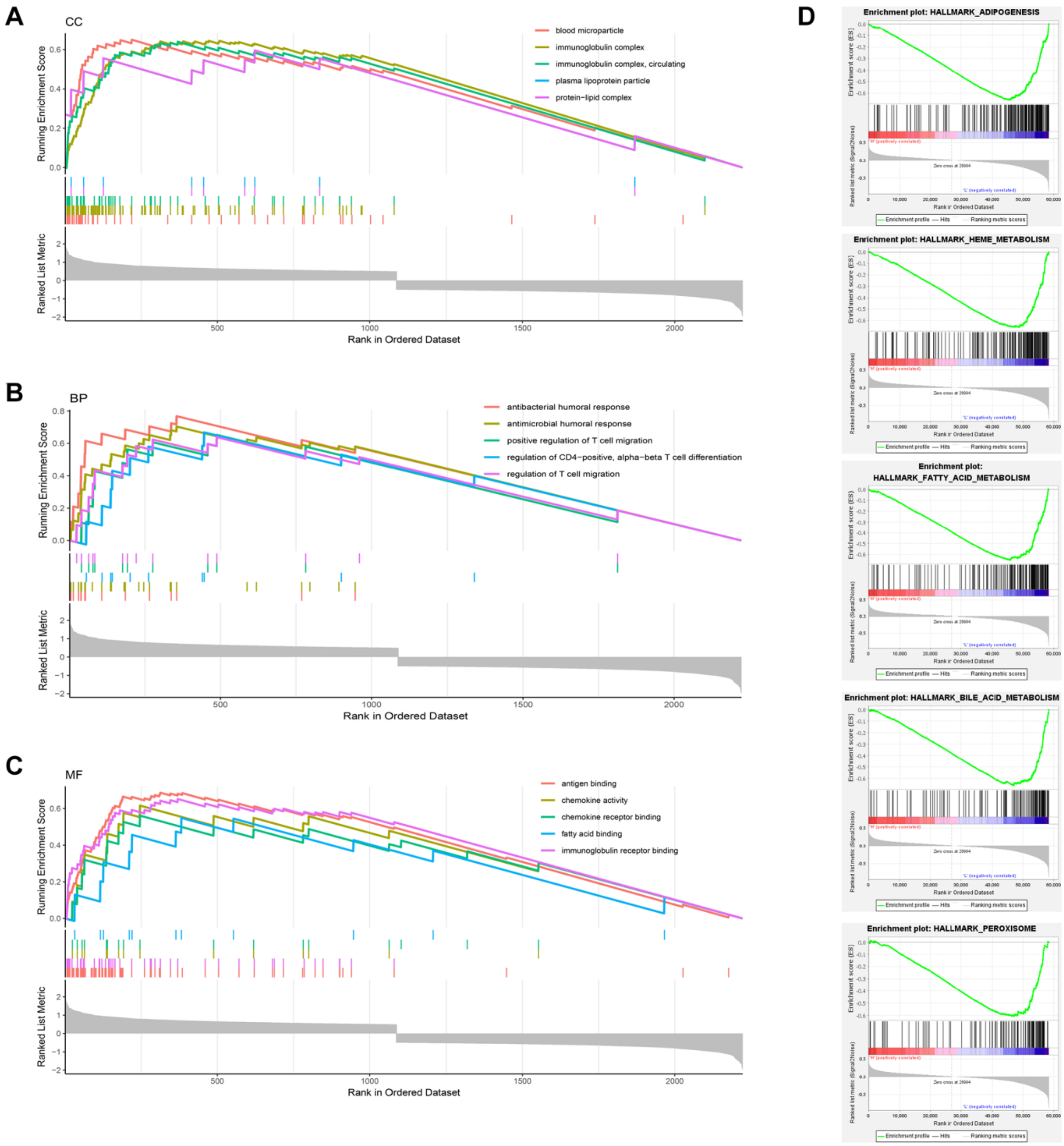

Supplementary Figure 8. GSEA analysis by corresponding software to show the significantly enriched cell components (**A**), Biological progression (**B**), Molecular function (**C**), and Hallmark pathways (**D**).

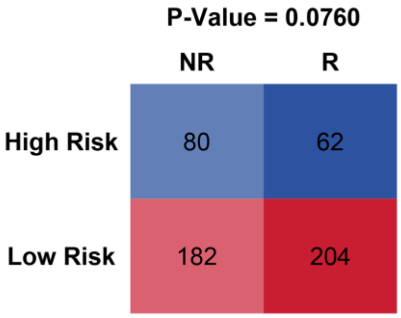

Supplementary Figure 9. Block chart shows the distribution number of responders (R) and non-responders (NR) in high- and low-risk groups.
